# Efficacy and Complications of Nasojejunal, Jejunostomy and Parenteral Feeding After Pancreaticoduodenectomy

**DOI:** 10.1007/s11605-012-1887-5

**Published:** 2012-04-20

**Authors:** Arja Gerritsen, Marc G. Besselink, Kasia P. Cieslak, Menno R. Vriens, Elles Steenhagen, Richard van Hillegersberg, Inne H. Borel Rinkes, I. Quintus Molenaar

**Affiliations:** 1 Department of Surgery, University Medical Center Utrecht, HP G04.228, P.O. Box 85500, 3508 GA Utrecht, The Netherlands; 2 Department of Dietetics, Julius Center for Health Sciences and Primary Care, University Medical Center Utrecht, Utrecht, The Netherlands

**Keywords:** Pancreas, Surgery, Feeding, Nutrition, Complications

## Abstract

**Background:**

European nutritional guidelines recommend routine use of enteral feeding after pancreaticoduodenectomy (PD) whereas American guidelines do not. Data on the efficacy and, especially, complications of the various feeding strategies after PD are scarce.

**Methods:**

Retrospective monocenter cohort study in 144 consecutive patients who underwent PD during a period wherein the routine post-PD feeding strategy changed twice. Patients not receiving nutritional support (n=15) were excluded. Complications were graded according to the Clavien-Dindo classification and the International Study Group of Pancreatic Surgery (ISGPS) definitions. Analysis was by intention-to-treat. Primary endpoint was the time to resumption of normal oral intake.

**Results:**

129 patients undergoing PD (111 pylorus preserving) were included. 44 patients (34%) received enteral nutrition via nasojejunal tube (NJT), 48 patients (37%) via jejunostomy tube (JT) and 37 patients (29%) received total parenteral nutrition (TPN). Groups were comparable with respect to baseline characteristics, Clavien ≥II complications (*P*=0.99), in-hospital stay (*P*=0.83) and mortality (*P*=0.21). There were no differences in time to resumption of normal oral intake (primary endpoint; NJT/JT/TPN: median 13, 16 and 14 days, *P*=0.15) and incidence of delayed gastric emptying (*P=*0.30). Duration of enteral nutrition was shorter in the NJT- compared to the JT- group (median 8 vs. 12 days, *P*=0.02). Tube related complications occurred mainly in the NJT-group (34% dislodgement). In the JT-group, relaparotomy was performed in three patients (6%) because of JT-leakage or strangulation leading to death in one patient (2%). Wound infections were most common in the TPN group (NJT/JT/TPN: 16%, 6% and 30%, *P*=0.02).

**Conclusion:**

None of the analysed feeding strategies was found superior with respect to time to resumption of normal oral intake, morbidity and mortality. Each strategy was associated with specific complications. Nasojejunal tubes dislodged in a third of patients, jejunostomy tubes caused few but potentially life-threatening bowel strangulation and TPN doubled the risk of infections.

## Introduction

Pylorus-preserving pancreaticoduodenectomy (PD) is the treatment of choice for (pre-)malignant neoplasms of the pancreatic head, ampulla, distal bile duct and duodenum.^1^ PD is associated with a relatively high morbidity rate, including a high incidence of delayed gastric emptying.^2^^–^ 4 The current guidelines of the European Society for Parenteral and Enteral Nutrition (ESPEN) recommend routine use of early enteral nutrition in case of patients undergoing major gastrointestinal surgery for cancer, including PD.^5^ In contrast, the current American Society of Parenteral and Enteral Nutrition (ASPEN) guidelines recommend postoperative nutritional support only in patients whom it is anticipated will be unable to meet their nutrient needs orally for a period of 7 to 10 days, which is not necessarily the case after PD.^6^

Specific evidence concerning the optimal feeding strategy after PD is scarce and hence the choice of feeding strategy depends mainly on individual preference. Although enteral and parenteral feeding after PD may be associated with complications, there are surprisingly little data available on this subject.

In our department, the preferred routine post-PD feeding strategy changed twice in the past 10 years; from jejunostomy tube feeding (JT) to total parenteral nutrition (TPN) to nasojejunal tube feeding (NJT). These changes were initiated by a perceived high rate of feeding-related complications associated with the JT and TPN feeding strategies as well as a lack of clear evidence in favour of any feeding technique. The aim of this study was to assess the efficacy and feeding-related complications of the various feeding strategies after PD in a single tertiary referral center.

## Methods

### Patients

A retrospective monocenter cohort study was performed in all 144 consecutive patients who underwent (pylorus preserving) PD at the University Medical Center Utrecht, between January 1st 2001 and December 30th 2010. Patients were categorised according to the feeding strategy decided upon prior to or during surgery: enteral feeding via NJT or JT, or TPN. Patients who could not be classified in these three groups or had no follow-up were excluded (*n* = 15). See the ‘Results’ section for details.

### Surgical Approach

PD was performed by a team specialised in hepatobiliary and pancreatic surgery. Reconstruction was typically performed with end-to-side duct-to-mucosa pancreatojejunostomy (ISGPS type IAS0),^7^ end-to-side hepaticojejunostomy and (antecolic) duodeno(gastro)jejunostomy.

### Routes of Nutrition

Enteral nutrition was delivered via either nasojejunal tube or jejunostomy tube. In the period January 2001–May 2010, Nutrison Standard was used and since May 2010 Nutrison Protein Plus (both from Nutricia, The Netherlands). In the NJT group, a nasojejunal tube (Freka Trelumina Tube, Fresenius Kabi Ltd, UK) was advanced for at least 30 cm through the duodenojejunostomy after the creation of the dorsal part of this anastomosis. In the JT group, a jejunostomy tube (Freka FCJ Set FR 9, Fresenius Kabi Ltd, UK) was advanced through the abdominal wall and into the bowel after the reconstruction phase of the pancreaticoduodenectomy. The tube was advanced for at least 30 cm and fixated to the bowel and the abdominal wall. In both the NJT and JT groups, enteral nutrition was started the first morning postoperatively at a rate of 25 ml/h and increased with 25 ml per day (2001–2009) or per 6 h (since May 2010) to the required amount as advised by the consulting dietitian.

TPN (NuTRIflex Lipid Special, B. Braun, Germany) was delivered via a central venous line. TPN was started the morning after surgery at a rate of 42 ml/h and increased with 500 ml per day to the required amount, according to dietitians’ advice.

In the NJT and JT groups, TPN was only given when enteral feeding was unsuccessful, but according to the intention-to-treat principle these patients remained in their assigned groups.

Oral intake was started on patient’s request and modulated depending on digestive symptoms. When oral intake exceeded 50 % of the daily required caloric intake, enteral or parenteral nutritional support was ceased. In the NJT group, the feeding tube was removed at this stage. In the JT group, the tube was only removed in the outpatient department 6 weeks postoperatively. Patients in the JT and TPN group received a nasogastric tube for gastric decompression only if necessary.

### Definitions

Postoperative pancreatic fistula, delayed gastric emptying and post-pancreatectomy haemorrhage were defined according to the International Study Group of Pancreatic Surgery (ISGPS) definitions.^8^^–^ 10 All complications were graded according to the Clavien–Dindo classification.^11^ The postoperative course was defined to be complicated if a complication occurred that required medical therapy or any form of intervention (Clavien–Dindo grade II or higher). Chyle leakage requiring very low fat elemental enteral nutrition was graded as Clavien–Dindo grade II, if there was no other indication for enteral nutrition (anymore). Infectious complications had to be confirmed by a positive culture result. Severe preoperative weight loss was defined as weight loss of 10 % or more within 6 months or 5 % or more within 1 month prior to surgery.

### Data Collection

Data were retrospectively collected from computerised clinical records. Baseline characteristics collected were patient demographics, body mass index (BMI), severe preoperative weight loss, indication for surgery, diagnosis, surgeon, type of surgery, operative time and blood loss.

Primary outcome was the time to resumption of normal oral intake, defined as the postoperative day on which intake was reported to be adequate by the treating physician or dietitian.

Secondary outcomes were time to start of oral and solid food intake, duration of (par)enteral nutrition, use of prokinetic agents, postoperative surgical, general and tube-related complications (in-hospital and during readmission), incidence of postoperative pancreatic fistula, delayed gastric emptying, post-pancreatectomy haemorrhage and chyle leakage, length of hospital stay, readmission within 30 days after discharge, relaparotomy and in-hospital mortality.

### Statistical Analysis

Analysis was by intention-to-treat. Values are expressed as median and interquartile range, unless specified otherwise. Data were analysed using SPSS for Windows version 15.0 (SPSS Inc., Chicago, IL, USA). Continuous non-normally distributed variables were compared using the Kruskal–Wallis or Mann–Whitney *U* test, and categorical variables were compared by chi-square or Fisher’s exact test as appropriate. For multivariable analysis, the binary logistic regression model was used. Statistical dependence between two non-parametric variables was assessed by Spearman correlation. A *P* value <0.05 was considered significant.

## Results

### Patients

Of the 144 patients who had undergone a (pylorus preserving) PD in the study period, 15 patients were excluded because they had no nutritional support (*n* = 9), received enteral nutrition via nasogastric tube (*n* = 4), underwent a modified surgical intervention (status after previous total gastrectomy, *n* = 1) or were transferred to another hospital (*n* = 1) leaving 129 patients eligible for further analysis.

Of these 129 patients, 44 (34 %) received enteral nutrition via NJT, 48 patients (37 %) via JT and 37 patients (29 %) received TPN. Baseline characteristics, including age, gender, BMI, severe preoperative weight loss, indication for surgery, diagnosis, procedure and blood loss did not differ between the groups (see Table 1). The three groups only differed in terms of the surgeon performing the procedure and the operative time.

**Table 1:** Baseline characteristics

	Nasojejunal tube	Jejunostomy tube	Total parenteral nutrition	P value
	(*n* = 44)	(*n* = 48)	(*n* = 37)	
Male	59%	69%	57%	0.47^d^
Age	63 (61–67)	65 (57–72)	66 (60–72)	0.34^c^
BMI	24.2 (22.0–26.7)	24.3 (22.8–25.9)	23.8 (22.0–26.9)	0.96^c^
Severe preoperative weight loss^a^	15 (34%)	14 (41%)	10 (34%)	0.79^d^
Indication				0.62^d^
Suspected malignancy	21 (48%)	33 (69%)	23 (62%)	
Proven malignancy	14 (32%)	11 (23%)	10 (27%)	0.29^e^
Other	9 (20%)	4 (8%)	4 (11%)	
Diagnosis				0.28^e^
Pancreatic adenocarcinoma	19 (43%)	23 (48%)	11 (30%)	
Ampullary adenocarcinoma	4 (9%)	11 (23%)	8 (22%)	
Cholangiocarcinoma	6 (14%)	3 (6%)	7 (19%)	
Benign	7 (16%)	3 (6%)	4 (11%)	
Other	8 (18%)	8 (17%)	7 (19%)	
Procedure				0.13^d^
Pylorus-preserving PD	34 (77%)	44 (92%)	29 (78%)	
Extended PD^b^	10 (23%)	4 (8%)	8 (22%)	
Intraoperative parameters				
Operative time (min)	370 (318–438)	291 (265–361)	367 (312–412)	0.001^c^
Blood loss (cc)	800 (500–1,350)	1,000 (550–1,500)	800 (575–1,500)	0.43^c^
RBC transfusion (U)	0 (0–0)	0 (0–2)	0 (0–1)	0.20^c^
FFP transfusion (U)	0 (0–0)	0 (0–0)	0 (0–0)	0.72^c^

*BMI* body mass index, *PD* pancreaticoduodenectomy, *RBC* red blood cells, *FFP* fresh frozen plasma

^a^17% missing data

^b^Whipple's PD (*n* = 5), PD + hemicolectomy (*n* = 3), PD + hemicolectomy + nefrectomy (*n* = 3), PD + hemicolectomy + portal vein (*n* = 2), PD + portal vein (*n* = 5), PD + partial corpus/cauda resection (*n* = 2), total pancreatectomy (*n* = 1), total pancreatectomy + portal vein (*n* = 1)

^c^Kruskal–Wallis test

^d^Chi-square test

^e^Fisher's exact test

### Efficacy

Time to resumption of normal oral intake (primary endpoint) did not differ between the three groups, with a median duration of 13 (10–19), 16 (13–24) and 14 (10–22) days in the NJT, JT and TPN group, respectively (*P* = 0.15). Duration of enteral nutrition was significantly shorter in the NJT group compared to the JT group [median 8 (6–12) vs. 12 (8–18) days, *P* = 0.02]. Time to start of oral intake was significantly shorter in the TPN-group, with a median duration of 5 (3–7), 5 (4–11) and 4 (2–5) days in the NJT, JT and TPN group, respectively (*P* = 0.02). All outcomes of nutritional and hospitalisation parameters are shown in Table 2.

**Table 2:** Nutritional and hospitalisation parameters

	Nasojejunal tube	Jejunostomy tube	Total parenteral nutrition	P value	P value
	(*n* = 44)	(*n* = 48)	(*n* = 37)		Enteral only^a^
Primary endpoint					
Time to resumption of normal oral intake (days)	13 (10–19)	16 (13–24)	14 (10–22)	0.15^c^	0.09^b^
Secondary endpoints					
Duration of enteral nutrition (days)	8 (6–12)	12 (8–18)	0 (0–0)		0.02^b^
Duration of parenteral nutrition (days)	0 (0–6)	0 (0–0)	9 (7–14)		0.06^b^
Time to start oral intake (days)	5 (3–7)	5 (4–11)	4 (2–5)	0.02^c^	0.11^b^
Time to start solid food intake (days)	9 (6–12)	12 (9–17)	9 (7–13)	0.10^c^	0.06^b^
Use of prokinetics (*n*)	12 (27%)	23 (48%)	14 (38%)	0.15^d^	0.05^d^
Length of hospital stay (days)	17 (12–23)	19 (14–24)	16 (13–27)	0.83^c^	0. 58^d^
Readmission within 30 days (*n*)	2 (4.5%)	3 (6.3%)	6 (16.2%)	0.19^e^	1.00^e^

^a^Nasojejunal vs jejunostomy tube

^b^Mann–Whitney *U* test

^c^Kruskal–Wallis test

^d^Chi-square test

^e^Fisher's exact test

### General Complications

Morbidity and mortality rates are shown in Table 3. Overall morbidity requiring therapy or intervention (Clavien–Dindo grade II or higher) and mortality did not differ between the groups.

**Table 3:** Postoperative complications

	Nasojejunal tube	Jejunostomy tube	Total parenteral nutrition	P value	P value
	(*n* = 44)	(*n* = 48)	(*n* = 37)		Enteral only^a^
Overall morbidity					
Clavien–Dindo grade I–V	37 (84%)	44 (92%)	34 (92%)	0.49^e^	0.26^d^
Clavien–Dindo grade ≥II	27 (61%)	30 (63%)	23 (62%)	0.99^d^	0.91^d^
Surgical morbidity^b^	36 (82%)	43 (90%)	33 (89%)	0.52^e^	0.29^d^
General morbidity^c^	20 (45%)	26 (54%)	17 (39%)	0.55^d^	0.40^d^
Tube-related morbidity	18 (41%)	11 (23%)	6 (16%)	0.03^d^	0.06^d^
Relaparotomy	7 (16%)	8 (17%)	7 (19%)	0.93^d^	0.92^d^
Number of procedures	19	15	13		
of which tube related	0	4	0		
Mortality	2 (4.5%)	6 (12.5%)	1 (2.7%)	0.21^e^	0.27^e^
of which tube related	0	1	0		

^a^Nasojejunal vs jejunostomy tube

^b^Including pancreatic fistula, delayed gastric emptying, postoperative haemorrhage, ileus, anastomotic bowel leak, chyle leak, biliary leak, fascial dehiscence, bowel ischaemia, enterocutaneous fistula, cholangitis, wound infection and intra-abdominal abscess

^c^Including line infection, urinary tract infection, pneumonia, deep venous thrombosis and pulmonary embolism

^d^Chi-square test

^e^Fisher's exact test

Surgical and general complication rates are listed in Table 4. The rates of clinically relevant postoperative pancreatic fistula (grade B/C, ISGPF), delayed gastric emptying (grade B/C, ISPGS) and postoperative haemorrhage (grade B/C, ISGPS) did not differ between the groups (overall rates 12 %, 42 % and 10 %, respectively).

**Table 4:** General and surgical morbidity

	Nasojejunal tube	Jejunostomy tube	Total parenteral nutrition	P value	P value
	(*n* = 44)	(*n* = 48)	(*n* = 37)		Enteral only^a^
Pancreatic fistula (grade B/C)	5 (11%)	6 (13%)	4 (11%)	1.00^c^	
Delayed gastric emptying (grade B/C)	15 (34%)	24 (50%)	15 (40%)	0.30^b^	
Postoperative haemorrhage (grade B/C)	6 (14%)	4 (8%)	3 (8%)	0.67^c^	
Ileus	2 (5%)	5 (10%)	0 (0%)	0.13^c^	
Anastomic bowel leak	1 (2%)	0 (0%)	0 (0%)	0.63^c^	
Chyle leak	9 (20%)	11 (23%)	2 (5%)	0.08^b^	
Biliary leak	5 (11%)	4 (8%)	3 (8%)	0.86^c^	
Fascial dehiscence	2 (5%)	0 (0%)	3 (8%)	0.13^c^	
Bowel ischaemia	1 (2%)	1 (2%)	0 (0%)	1.00^c^	
Enterocutaneous fistula	1 (2%)	0 (0%)	1 (3%)	0.53^c^	
Other surgical complications	3 (7%)	2 (4%)	2 (5%)	0.89^c^	
Infections					
Cholangitis	3 (7%)	0 (0%)	2 (5%)	0.22^c^	
Wound infection	7 (16%)	3 (6%)	11 (30%)	0.02^b^	0.19^c^
Intra-abdominal abscess	3 (7%)	1 (2%)	2 (5%)	0.58^c^	
Line infection	8 (18%)	7 (15%)	3 (8%)	0.42^b^	
Urinary tract infection	6 (14%)	6 (13%)	3 (8%)	0.79^c^	
Pneumonia	9 (20%)	8 (17%)	4 (11%)	0.50^b^	
Deep venous thrombosis	1 (2%)	1 (2%)	0 (0%)	1.00^c^	
Pulmonary embolism	3 (7%)	1 (2%)	2 (5%)	0.58^c^	
Other general complications	10 (23%)	12 (25%)	14 (32%)	0.27^b^	

^a^Nasojejunal vs jejunostomy tube, only reported when overall *P* value <0.05

^b^Chi-square test

^c^Fisher's exact test

### Feeding-Related Complications

Tube-related complications were more common in the NJT group as compared to the JT group (41 % vs. 23 %, *P* = 0.06). This difference was mainly caused by a 34 % (*n* = 15) dislodgement rate of the intraoperatively placed nasojejunal tubes (Table 5). These dislodgements occurred after a median of 7 (5–12) days. Tubes were replaced in eight of the 15 patients. The complications in the JT group tended to be more severe, including leakage and torsion of the small bowel around the tube, requiring four relaparotomies in three patients (6 %) and eventually leading to death in one patient (2 %), due to multi-organ failure caused by small bowel ischaemia. The rate of wound infections was significantly higher in the TPN group (NJT/JT/TPN = 16 %, 6 % and 30 %, *P* = 0.02).

**Table 5:** Tube-related morbidity

	Nasojejunal tube	Jejunostomy tube	Total parenteral nutrition	P value	P value
	(*n* = 44)	(*n* = 48)	(*n* = 37)		Enteral only^a^
Dislodgement of primary placed tube	15 (34%)	4 (8%)	3 (8%)	0.001^d^	0.002^d^
Disabling blockage	4 (9%)	5 (10%)	0 (0%)	0.11^e^	1.00^e^
Infection of feeding tube/central venous line^b^	0 (0%)	1 (2%)	3 (8%)	0.10^e^	1.00^e^
Haemorrhage (not requiring transfusion)	0 (0%)	2 (4%)	0 (0%)	0.33^e^	0.50^e^
Other^c^	0 (0%)	5 (10%)	0 (0%)	0.01^e^	0.06^e^
Tube-related relaparotomy	0 (0%)	3 (6%)	0 (0%)	0.11^e^	0.24^e^
Tube-related mortality	0 (0%)	1 (2%)	0 (0%)	1.00^e^	1.00^e^

^a^Nasojejunal vs jejunostomy tube

^b^See Table 4 for all infectious complications

^c^Small bowel leakage (*n*=2), bowel torsion (*n*=2), subcutaneous emphysema and fluid collection around insertion site (*n*=1)

^d^Chi-square test

^e^Fisher's exact test

### Multivariable Analysis

The patient volume increased during the 10-year study period. Average annual volume in the first 3 years was 5 versus 25 in the last 3 years (*P* = 0.001). Therefore, year of procedure was entered as a variable in a multivariable logistic regression analysis that also adjusted for differences in age, gender, BMI and surgeon. After adjustment, there still was no difference between the three feeding strategies in the rate of morbidity or mortality.

Age and gender were found to be an independent factor influencing morbidity (Clavien–Dindo grade II or higher). A 1-year increase in age had an OR of 1.05 in developing a complication (*P* = 0.02). The male gender had an OR of 2.48 in developing a complication compared to the female gender (*P* = 0.02). No independent factors for mortality were found.

## Discussion

This is the largest comparative study to date on efficacy and complications of jejunostomy, nasojejunal and total parenteral feeding after PD. None of the feeding strategies was found superior with respect to time to resumption of normal oral intake, morbidity and mortality. Each strategy was associated with specific complications. Nasojejunal tubes dislodged in a third of patients, jejunostomy tubes caused few but potentially life-threatening bowel strangulation and TPN doubled the risk of wound infections. Although these complications varied widely in both incidence and severity, it seems that if feeding is desired a nasojejunal tube is the feeding strategy of choice, as replacement of a nasojejunal tube (although frequently required) is to be preferred over infections and bowel strangulation.

Only one study previously compared the efficacy and safety of different routes of enteral nutritional support after PD.^12^ This monocenter cohort study analysed 100 patients receiving enteral nutrition via either percutaneous transperitoneal jejunostomy, gastrojejunostomy or nasojejunal tube. This study did not use ISGPS definitions or multivariable analysis to account for confounders such as surgeon’s preference. As in our study, no significant differences were observed between the groups in time to resumption of normal oral intake, as well as in hospital stay, while duration of feeding was significantly shorter in NJT patients. Tube related complications, however, were observed less frequently than in our study. Dislodgement of the NJT was seen in only 5 % of patients, as compared to 34 % in our study. The authors stated this relatively low dislodgement rate to be caused by the use of a tube with a wider diameter (10/8 French), enabling more secure fixation at the nostrils. The tubes used in our study, however, have an even larger diameter (16/9 French) due to the three lumina. In the general feeding literature, a dislodgement rate of NJTs of 16–36 % has been reported.^13^^–^ 17 Several techniques have been described to prevent inadvertent NJT dislodgement. One of the most successful ones is nasal bridling.^18^ In a randomised controlled trial (*n* = 80), comparing the nasal bridle technique with an adhesive tape device, dislodgement rate was reduced from 63 % in the unbridled to 18 % in the bridled group, with only few and minor adverse events.^19^ Another technique is the clipping of the tip of the nasojejunal tube to the bowel mucosa,^20^ but this is impractical during laparotomy.

Although rare, bowel strangulation and leakage are well-known, potentially lethal complications of percutaneous jejunostomy tubes. In a series of 2,022 patients undergoing laparotomy for mostly complex upper-abdominal operations, jejunostomy resulted in 34 tube-related complications in 29 patients (1.5 %).^21^ The most common complication was occlusion or dislodgement in 15 patients (0.7 %), and the most serious complication was bowel necrosis in three patients (0.2 %), leading to death in two patients. Intestinal occlusion and volvulus was described in three patients (0.2 %), leading to death in one and intra-abdominal infections in three other (0.2 %). A literature review in 1,788 patients with jejunostomy tubes found a strangulation rate of 0.3 % and an intra-abdominal infection rate of 0.8 %.^21^

Increased risk of infections with the use of parenteral nutrition is also well known. A meta-analysis by Braunschweig et al., combining 27 randomised controlled trials (*n* = 1,827), found a significantly increased risk of infections with parenteral compared to enteral nutrition (RR 0.64),^22^ corresponding with the results of a previous study in patients after PD.^23^

Duration of enteral nutrition was found to be significantly shorter in the NJT group compared to the JT group (median 8 vs. 12 days). This can be explained by the fact that enteral feeding in the NJT group was often interrupted due to tube dislodgement, and attempts to stop enteral nutrition were undertaken earlier to stimulate oral intake and relief patients of their tube before discharge.

The strength of the current study lies in the use of the generally accepted ISGPS definitions and the Clavien–Dindo classification for postoperative complications, making comparison of data more reliable. It is known that the use of the ISGPS definitions results in a relatively high incidence of complications.^3^ The main limitation of this study is its retrospective design and therefore nonrandom (but rather chronological) allocation of patients into the different feeding strategies. Furthermore, during the study period the annual volume of PD increased. However, as there was no relation between the studies primary endpoint (time to resumption of normal oral intake) and surgeon (*P* = 0.50) or year of surgery (*P* = 0.21), we feel the impact of these confounders is small. The group of patients with an oral diet after PD was too small and heterogeneous for reliable conclusions and hence not included in the analysis.

Interestingly, several large studies found good results with a normal oral diet (without routine nutritional support) after PD. Yermilov et al. reviewed the California Cancer Registry (1994–2003) for outcomes of 1,873 patients who underwent PD for adenocarcinoma receiving either parenteral feeding (14 %), jejunostomy tube feeding (23 %) or an oral diet without supplemental nutritional support (63 %). This study did not include data on nasojejunal feeding. They showed a significantly shorter length of hospital stay in the normal diet cohort.^24^ Martignoni et al. prospectively studied a cohort of 64 patients and reported, besides an increase in length of stay, a significantly higher prevalence of delayed gastric emptying in patients with enteral nutrition, compared to patients with an oral diet.^25^ In contrast to these studies, two other studies suggest that routine enteral nutrition is better than ‘standard care’. In a randomised controlled trial (*n* = 36) by Mack et al., length of hospital stay was reduced by routine gastrojejunostomy tube feeding as compared to ‘standard care‘ after PD.^26^ This study did not define ‘standard care’. Baradi et al. retrospectively studied patients with postoperative nasojejunal or gastrojejunal tube feeding or an oral diet after PD (*n* = 180). Enteral feeding was associated with significantly less use of TPN and lower rates of readmission and complications. Length of stay did not differ between the two groups.^27^

If an oral postoperative diet is used, what strategy should be followed? A recent review suggested that implementation of a fast-track perioperative pathway [or enhanced recovery after surgery (ERAS) program] in pancreatic surgery could lead to reduced hospital stay and reduced costs without an increase in morbidity, mortality or re-admission rates.^28^

The discrepancy between current European ‘routine’ and American ‘on demand’ guidelines and the feeding related complications described in this study support use of the American ‘on demand’ nutrition guidelines. One could argue that patients should be started on a regular oral diet as soon as possible after PD as is current practice after most other major surgeries. Only in case of severe preoperative weight loss or a complicated postoperative course (such as pancreatic fistula),^29^ enteral nutrition should be started. This ‘on demand’ strategy would prevent many patients from the discomfort of a feeding tube/line and would save the additional costs of enteral feeding. Future randomised studies should test this hypothesis by comparing outcomes of a routine oral diet (with on demand nasojejunal feeding) with routine nasojejunal feeding after PD.

## References

[1] Hidalgo M (2010). Pancreatic cancer. N Engl J Med..

[2] Malleo G, Crippa S, Butturini G, Salvia R, Partelli S, Rossini R (2010). Delayed gastric emptying after pylorus-preserving pancreaticoduodenectomy: validation of International Study Group of Pancreatic Surgery classification and analysis of risk factors. HPB (Oxford)..

[3] Tan WJ, Kow AW, Liau KH (2011). Moving towards the New International Study Group for Pancreatic Surgery (ISGPS) definitions in pancreaticoduodenectomy: a comparison between the old and new. HPB (Oxford)..

[4] Welsch T, Borm M, Degrate L, Hinz U, Büchler MW, Wente MN (2010). Evaluation of the International Study Group of Pancreatic Surgery definition of delayed gastric emptying after pancreatoduodenectomy in a high-volume centre. Br J Surg..

[5] Weimann A, Braga M, Harsanyi L, Laviano A, Ljungqvist O, Soeters P (2006). ESPEN Guidelines on enteral nutrition: surgery including organ transplantation. Clin Nutr..

[6] ASPEN Board of Directors and the Clinical Guidelines Task Force (2002). Guidelines for the use of parenteral and enteral nutrition in adult and pediatric patients. JPEN J Parenter Enteral Nutr.

[7] Shukla PJ, Barreto SG, Fingerhut A, Bassi C, Büchler MW, Dervenis C (2010). Toward improving uniformity and standardization in the reporting of pancreatic anastomoses: a new classification system by the International Study Group of Pancreatic Surgery (ISGPS). Surgery..

[8] Bassi C, Dervenis C, Butturini G, Fingerhut A, Yeo C, Izbicki J (2005). International Study Group on Pancreatic Fistula Definition. Postoperative pancreatic fistula: an international study group (ISGPF) definition. Surgery..

[9] Wente MN, Bassi C, Dervenis C, Fingerhut A, Gouma DJ, Izbicki JR (2007). Delayed gastric emptying (DGE) after pancreatic surgery: a suggested definition by the International Study Group of Pancreatic Surgery (ISGPS). Surgery..

[10] Wente MN, Veit JA, Bassi C, Dervenis C, Fingerhut A, Gouma DJ (2007). Postpancreatectomy hemorrhage (PPH): an International Study Group of Pancreatic Surgery (ISGPS) definition. Surgery..

[11] Dindo D, Demartines N, Clavien PA (2004). Classification of surgical complications: a new proposal with evaluation in a cohort of 6336 patients and results of a survey. Ann Surg..

[12] Abu-Hilal M, Hemandas AK, McPhail M, Jain G, Panagiotopoulou I, Scibelli T, Johnson CD, Pearce NW (2010). A comparative analysis of safety and efficacy of different methods of tube placement for enteral feeding following major pancreatic resection. A non-randomized study. JOP.

[13] Metheny NA, Schnelker R, McGinnis J (2005). Indicators of tube site during feedings. J Neurosci Nurs..

[14] Wiggins TF, DeLegge MH (2006). Evaluation of a new technique for endoscopic nasojejunal feeding-tube placement. Gastrointest Endosc..

[15] Brandt CP, Mittendorf EA (1999). Endoscopic placement of nasojejunal feeding tubes in ICU patients. Surg Endosc..

[16] Mahadeva S, Malik A, Hilmi I (2008). Transnasal endoscopic placement of nasoenteric feeding tubes: outcomes and limitations in non-critically ill patients. Nutr Clin Pract..

[17] Boulton-Jones JR, Lewis J, Jobling JC (2004). Experience of post-pyloric feeding in seriously ill patients in clinical practice. Clin Nutr..

[18] McGuirt WF, Strout JJ (1980). “How I do it”: head and neck. A targeted problem and its solution: securing of intermediate duration feeding tubes. Laryngoscope..

[19] Seder CW, Stockdale W, Hale L, Janczyk RJ (2010). Nasal bridling decreases feeding tube dislodgment and may increase caloric intake in the surgical intensive care unit: a randomized, controlled trial. Crit Care Med..

[20] Schrijver AM, Siersema PD, Vleggaar FP, Hirdes MM, Monkelbaan JF (2011). Endoclips for fixation of nasoenteral feeding tubes: a review. Dig Liver Dis..

[21] Myers JG, Page CP, Stewart RM, Schwesinger WH, Sirinek KR, Aust JB (1995). Complications of needle catheter jejunostomy in 2,022 consecutive applications. Am. J. Surg.

[22] Braunschweig CL, Levy P, Sheean PM, Wang X (2001). Enteral compared with parenteral nutrition: a meta-analysis. Am J Clin Nutr..

[23] Gianotti L, Braga M, Gentilini O, Balzano G, Zerbi A, Di Carlo V (2000). Artificial nutrition after pancreaticoduodenectomy. Pancreas..

[24] Yermilov I, Jain S, Sekeris E, Bentrem DJ, Hines OJ, Reber HA (2009). Utilization of parenteral nutrition following pancreaticoduodenectomy: is routine jejunostomy tube placement warranted?. Dig Dis Sci..

[25] Martignoni ME, Friess H, Sell F, Ricken L, Shrikhande S (2000). Enteral nutrition prolongs delayed gastric emptying in patients after Whipple resection. Am J Surg..

[26] Mack LA, Kaklamanos IG, Livingstone AS, Levi JU, Robinson C, Sleeman D (2004). Gastric decompression and enteral feeding through a double-lumen gastrojejunostomy tube improves outcomes after pancreaticoduodenectomy. Ann Surg..

[27] Baradi H, Walsh RM, Henderson JM, Vogt D, Popovich M (2004). Postoperative jejunal feeding and outcome of pancreaticoduodenectomy. J Gastrointest Surg.

[28] Ypsilantis E, Praseedom RK (2009). Current status of fast-track recovery pathways in pancreatic surgery. JOP..

[29] Klek S, Sierzega M, Turczynowski L, Szybinski P, Szczepanek K, Kulig J (2011). Enteral and parenteral nutrition in the conservative treatment of pancreatic fistula: a randomized clinical trial. Gastroenterology..

